# Examining Racial Identity Invalidation, Curvy Ideal Internalization, and Maladaptive Orthorexia Symptoms in Black–White Multiracial Women

**DOI:** 10.3390/bs16091562

**Published:** 2026-09-03

**Authors:** Sophie Pringle, Jennifer B. Webb, Mariah Roseboro

**Affiliations:** 1 Department of Psychological Science, Health Psychology Ph.D. Program, University of North Carolina at Charlotte, Charlotte, NC 28223, USA; springl2@charlotte.edu; 2 Department of Psychology, Counseling Psychology Ph.D. Program, Virginia Commonwealth University, Richmond, VA 23284, USA; roseboromj@vcu.edu

**Keywords:** orthorexia, multiracial, multiracial discrimination, curvy ideal

## Abstract

Black–White (B/W) multiracial women often experience racial identity invalidation (RII) due to holding races of opposite social status. Additionally, they may experience Eurocentric thinness pressures, while simultaneously navigating curvier body ideals valued in Black communities. B/W multiracial women’s eating and embodiment remains largely under-researched. This study examined associations between RII, curvy ideal idealization (CII), and maladaptive orthorexia symptoms (MOSs), in addition to the indirect effects of RII on MOSs via CII in a sample of B/W multiracial cisgender women, *N* = 290 (*M_Age_* = 34; range = 18–61). Participants completed an online survey through Prolific assessing demographics, RII, MOSs, and CII. Pearson’s correlations demonstrated significant positive relationships between MOSs and RII distress, RII frequency, and CII—global; RII distress and CII—global, thick/curvy, and breast size; and RII frequency and CII—breast size. No significant associations emerged between RII frequency and CII—global, thick/curvy, and thin waist or RII distress and CII—thin waist. The indirect effect of RII distress on MOS via CII—global was the only significant pathway (β = 0.04, 95% CI: 0.01 to 0.07). The results call for the need to consider the internalization of opposing cultural body ideals and the experience of discriminatory stressors as potential clinically relevant sociocultural factors associated with orthorexic eating patterns in B/W multiracial women.

## 1. Introduction

Multiracial individuals represent one of the fastest-growing demographics in the United States, in which it is estimated that 20% of the US population will identify as multiracial by 2050 (Ream, 2023), a 9.8% increase from 2010 (10.2%). There is significant heterogeneity in the multiracial population; however, multiracial individuals frequently experience anti-Black and multiracial discrimination (Butler et al., 2025). For instance, Black–White (B/W) multiracial individuals often experience racial identity invalidation or the rejection of their multiracial identity due to the historical one-drop rule (i.e., individuals with Black ancestry are identified as Black to keep Whiteness pure) (Reyna, 2022). Additionally, B/W multiracial women experience “double jeopardy” because they hold multiple marginalized identities (e.g., Black and a woman), putting them at a higher risk of negative health outcomes, such as disordered eating (Burke et al., 2021; Oh et al., 2023; Piran, 2016; Spates et al., 2020; Van Dyne et al., 2023). Identifying as a woman and holding identities of multiple racial groups may increase these health concerns by experiencing conflicting body ideals (Burke et al., 2020; Burke et al., 2021). For example, B/W multiracial women may simultaneously experience Eurocentric pressures of thinness in tandem with the contrasting preference for curvier bodies valued in Black communities. Moreover, in efforts to cope with these embodiment-related stressors, they may be vulnerable to endorsing contemporary maladaptive orthorexia symptoms, referring to the overvaluing and preoccupation with “healthy” eating (Barrada & Roncero, 2018). Notably, there remains a lack of scholarship evaluating these relationships in the multiracial population. Therefore, the current study investigated the indirect effects of racial identity invalidation on maladaptive orthorexia symptoms via curvy ideal internalization and examined the relationship among these variables in B/W multiracial women in the U.S. This overarching aim is responsive to recent calls by experts in the body image and embodiment field for the critical need to center the intersectional experiences of under-researched minoritized populations such as multiracial individuals through a bioecological lens on risk and resilience (Landor et al., 2024; Lowy et al., 2021; Ramseyer Winter et al., 2026).

### 1.1. Racial Identity Invalidation, Minority Stress, and Health

Racial identity invalidation is the denial, misperception, or rejection of one’s multiracial identity from both White and racial minority groups (Franco & O’Brien, 2018). Appearance-based identity invalidation may be particularly salient for B/W multiracial women as they report higher levels of phenotype validation compared to multiracial men, in which the intersection of sexism and racism may play a role (Franco & O’Brien, 2018; Rockquemore & Brunsma, 2002). Research suggests that these forms of oppression put multiracial women at a higher risk of adverse mental health outcomes (Oh et al., 2023), with Black multiracial individuals showing some of the highest rates of poor health within the multiracial population (Choi et al., 2025).

The minority stress theory (Meyer, 2003) suggests that discrimination, stigma, and marginalization-related stress associated with one’s minority status can lead to higher rates of negative health outcomes. Therefore, the minority stress theory (Meyer, 2003) recognizes racial identity invalidation as a minority-related stressor contributing to adverse health outcomes, such as poorer self-esteem, an increased risk of body image concerns, and disordered eating among multiracial groups (Convertino et al., 2021; Franco & O’Brien, 2018; Rodgers et al., 2025). Research suggests that racial/ethnic identification plays an important role in women of color’s body image experiences, in which racial identity invalidation is a critical factor. B/W multiracial women frequently encounter appearance-based racial invalidation from both White and Black groups based on how they phenotypically present (e.g., skin tone, hair type, etc.) (Rockquemore & Brunsma, 2004). In turn, B/W multiracial women often identify as Black because it is how others perceive them, yet they are frequently rejected by their monoracial Black peers (Goodwin, 2026). Additionally, previous studies suggest that B/W multiracial individuals raised in predominantly White areas have higher odds of body image concerns (Keigan et al., 2024). Thus, B/W multiracial women may feel like they do not belong in either racial group, contributing to racial identity and body concerns (Eid & Parker, 2023). Moreover, multiracial individuals often shift their racial identification based on their physical appearance or the degree to which they try to fit in with peers (Zamora & Padilla, 2024). This suggests that B/W multiracial women are often shifting their identity based on the salient sociocultural contexts in which they are embedded. Therefore, they may also experience increased susceptibility to endorsing culturally specific appearance ideals related to their racialized identity.

Lower racial/ethnic identification has been linked to increased influence by Western media and Eurocentric pressures in Black women (Murray et al., 2025). Conversely, Black and Black biracial women with greater racial/ethnic identification are more likely to accept their non-Eurocentric physical features and are less likely to internalize Eurocentric ideals and experience body dissatisfaction (Keigan et al., 2024). Additionally, Black and Black biracial women that endorse a strong racial/ethnic identification are associated with higher thick/curvy internalization (Keigan et al., 2024). This suggests that B/W multiracial women who experience appearance-based racial identity invalidation may feel pressured to identify with and endorse common body ideals associated with their Black identity. The previous literature indicates that negative body image in people of color is greatly associated with culturally specific appearance ideals rather than Western ideals focused on thinness (Landor et al., 2024). Thus, they may begin internalizing body standards connected to the Black community such as the curvy/thick ideal to affirm their racial identification as a Black woman. Furthermore, this lends to the importance of recognizing various sociocultural contexts and experiences, such as racial identity invalidation, that may be associated with B/W multiracial women’s endorsement of the curvy/thick ideal and eating behaviors to maintain this ideal. This pioneering conceptual framework is aligned with more contemporary sociocultural models that acknowledge the role of structural oppression in undermining body image, embodiment, and eating behavior in marginalized populations (Landor et al., 2024; Ramseyer Winter et al., 2026; Rodgers et al., 2025).

Intersectionality, referred to as the interlocking systems of power and marginalization related to the intersection of multiple identities (Crenshaw, 1989), is essential in understanding how the mixed and complex identities of B/W multiracial women likely contribute to higher rates of eating disorders among multiracial individuals. Their intersecting identities of multiple races of opposite social status and gender contribute to B/W multiracial women’s risk of experiencing racial identity invalidation (Franco & O’Brien, 2018). In turn, this contributes to the development and maintenance of adverse health concerns, such as disordered eating due to experiencing conflicting body ideals and negative embodiment (Burke et al., 2020; Burke et al., 2021; Franco & O’Brien, 2018). Therefore, intersectionality can provide a powerful framework for disordered eating and embodiment research in recognizing B/W multiracial women’s concurrent membership in different groups (Burke et al., 2020; Rodgers et al., 2025).

### 1.2. Embodiment, Curvy Ideal Internalization, and Orthorexia

Embodiment is the experience and understanding of the world through our bodies’ lived experiences. It comprises five dimensions: body–self connection, agency, desire, self-attunement, and resisting objectification (Piran, 2017; Piran et al., 2020). The Developmental Theory of Embodiment (DTE; Piran & Teall, 2012) acknowledges both negative and positive sociocultural experiences in physical, mental, and social domains that influence one’s quality of embodiment and, in turn, one’s health across the lifespan. For example, B/W women may encounter frequent racial discrimination (e.g., racial identity invalidation) and receive critical messages from society related to their appearance characteristics (Piran, 2017). This is associated with a disruption in body–self connection and perceiving one’s body from an observer’s perspective. In turn, this is linked to disrupted embodied experiences, disordered eating practices, and negative body image (Burychka et al., 2021; Piran, 2017). In fact, in a separate analysis, the authors observed a significant indirect effect of racial identity invalidation on mindful eating via positive embodiment among B/W multiracial women (Pringle et al., 2026).

Moreover, Black multiracial women lie at the intersection of two cultures in which they may believe they need to adhere to the beauty ideals of both mainstream White culture (e.g., the thin ideal) and Black culture (e.g., thick/curvy ideal). The curvy ideal is commonly endorsed among women of color (Walker et al., 2022). The curvy ideal can be described as “thick,” referring to a larger lower body, such as a large butt, hips, and thighs, or “slim–thick,” denoting an hourglass-shaped body with large breasts, hips, and butt, but a slim waist (Walker et al., 2022). Notably, women of color’s body ideals are often context-specific, in which thick and slim–thick ideals are common among them; however, they may shift to thinner ideals when they are immersed in White-dominated spaces (Walker et al., 2022). This further suggests that B/W multiracial women may be at increased risk of experiencing various body ideal pressures. Women who feel pressured to conform to the body ideals of two cultures are at an increased risk of eating disorders and maladaptive exercise behaviors (Chilson et al., 2025). In turn, B/W multiracial women may have higher odds of developing body dissatisfaction and vulnerability to endorsing maladaptive orthorexia symptoms.

Low self-concept clarity (lack of a clear sense of one’s identity) is linked to increased vulnerability to negative body image (e.g., body dissatisfaction) due to increased tendency to engage in appearance-based comparisons (Carter & Vartanian, 2022). Appearance-focused media has been increasingly linked to body image issues among women based on exposure to idealized body ideals (Gahler et al., 2023b). B/W multiracial women may experience increased appearance-based comparisons as a way of making sense of their racial identity (Chan, 2017). Thus, they may encounter diverse appearance ideals. A recent study explored exposure to thin and curvy models via Instagram and its association with girls’ willingness to engage in behaviors to be thinner or curvier and found that exposure to different idealized body types was associated with greater preference for that body type and body-change motivations respective of the corresponding thin or curvy ideal (Ferdousi et al., 2023). This suggests that B/W multiracial women may encounter various body ideals and sociocultural pressures related to the prevalent appearance standards they are commonly surrounded by. Notably, the findings also indicate that, while a strong racial/ethnic identification is associated with higher thick/curvy internalization (Keigan et al., 2024), it is important to recognize that endorsing a curvy ideal does not necessarily act as a protective or harmless alternative to the thin ideal; rather, exposure to different body ideals may be associated with different methods of maladaptive body-altering behaviors in which the processes underlying these effects may be similar (Ferdousi et al., 2023).

Another study that examined the associations between Instagram use, body image outcomes, and social media internalization of appearance ideals (thin to curvy) in a diverse sample of women revealed that, for Black women, time spent on Instagram was linked to greater social internalization of appearance ideals (Gahler et al., 2023a). The results also revealed that greater social internalization of appearance ideals was associated with more body dissatisfaction, less body appreciation, and a desire to be curvier, compared to the desire to be thin as endorsed by Asian-American women. These findings suggest that appearance-based internalization is a sociocultural process that is influenced by the specific body ideal being socially reinforced (e.g., seeing those with a specific body type while engaging with media forms). The results further indicate that increased appearance-based comparisons are connected to negative body image.

Women with a more negative body image recall memories that are characterized by more negative body appraisals (Von Spreckelsen et al., 2023), in which B/W multiracial women with low self-concept who experience racial identity invalidation based on physical appearance may be more likely to recall these memories when they are linked to negative body appraisals. Moreover, consistent with the DTE (Piran & Teall, 2012), B/W multiracial women may recall moments of social exclusion during encounters of appearance racial identity invalidation if they do not fit the idealized appearance standard (Piran, 2017). Thus, they may begin to view themselves from the observer’s perspective and internalize the culturally specific appearance ideal linked to their perceived racial identity. In turn, B/W multiracial women may endorse maladaptive eating practices such as orthorexia to navigate their weight and shape concerns, and modify their body to fit the idealized standard.

Orthorexia can be understood as an obsession with healthy food, food quality (e.g., “clean eating”), and proper nutrition to avoid ill health and diseases, to maximize one’s physical health, and to achieve a morally superior feeling of perfection and purity (Bratman, 1997). It can be described as “healthy” orthorexia symptoms, regarding “pursuing a “healthy” diet,” or maladaptive orthorexia symptoms, referring to the overvaluing of and preoccupation with healthy eating (Barrada & Roncero, 2018). Higher levels of orthorexia symptoms are linked to more compulsive exercise, weight and shape concerns, and body dissatisfaction (Zohar et al., 2023). In a recent study, Scheiber et al. (2023) examined the association between social media and orthorexia tendencies in addition to whether thin-ideal internalization, muscular internalization, appearance comparison, and body dissatisfaction were mechanisms linked to orthorexia symptoms. They found that participants’ engagement with health and fitness accounts on social media was positively related to orthorexia tendencies. Additionally, greater involvement with health and fitness accounts on social media was associated with higher thin-ideal and muscular internalizations and in turn contributed to higher orthorexia symptoms. More significant engagement in social media was also linked to higher appearance comparisons, and the authors suggested that this may lead to higher body dissatisfaction. These findings indicate that sociocultural pressures related to appearance ideal internalization are significant mechanisms related to orthorexia symptoms. This is consistent with the DTE (Piran & Teall, 2012), which acknowledges sociocultural factors that influence B/W multiracial women’s quality of embodiment. For example, the DTE (Piran & Teall, 2012) recognizes that women’s body journeys are influenced by social constructions or expectations that regulate the embodied lives of individuals. Thus, increased pressure to comply with objectifying appearance expectations (e.g., contexts with a salient thin ideal, curvy ideal, etc.) may reduce experiences of a B/W multiracial woman’s embodied comfort and agency, which may result in extensive engagement in body practices aimed at altering appearance, such as orthorexic eating behaviors (Piran, 2017).

It is also important to recognize that these idealized appearance expectations are often unrealistic to obtain and maintain, which in turn may contribute to increased orthorexia symptoms. Di Gesto et al. (2022) examined the effects of Instagram likes and disclaimer labels (altered appearance of photo) on self-awareness, body dissatisfaction, and social physique anxiety among Italian women and found that participants exposed to an Instagram image that was highly socially endorsed (high number of likes) reported higher levels of both body dissatisfaction and social physique anxiety compared to those with few likes. Notably, these findings remained when controlling for women’s internalization levels. The results suggest that a high number of likes represents the endorsement of idealized appearance, in which more likes signify social acceptance/approval of a specific aesthetic. These frequent social comparisons based on physical appearance are associated with body dissatisfaction, suggesting that sociocultural pressures to endorse unrealistic aesthetic standards may be a critical factor in conceptualizing the relationship between racialized appearance invalidation, internalized body ideals, and body-modifying eating practices. Moreover, consistent with the DTE (Piran & Teall, 2012), feeling pressure to comply with aesthetic standards may disrupt a woman’s embodied agency and comfort in addition to yielding to unrealistic ideal appearance-related objectification. Given this, women may engage in maladaptive eating practices such as orthorexia in an attempt to alter their body to fit the internalized, socially reinforced body ideal. This is also consistent with the previous literature that suggests that the desire for the curvy and slim–thick ideal is associated with weight and shape concerns, food preoccupation and restriction, and eating shame (Hernández et al., 2021; McComb & Mills, 2022a).

Furthermore, Messer et al. (2022) examined the prospective associations between five components of body image (i.e., overvaluation, dissatisfaction, preoccupation, bodychecking, and body image avoidance) and orthorexia nervosa symptoms in a sample of diverse women and found that an overvaluation of weight and shape concerns uniquely predicted orthorexia nervosa symptoms over time. The authors suggest that judgments of self-worth contingent upon weight and shape are unique features of negative body image that may influence symptoms of orthorexia nervosa. Moreover, given that women who aspire to the thin or slim–thick ideal report greater overall disordered eating, dietary restraint, eating, shape and weight concerns, appearance ideal internalization, body image investment, and physical appearance perfectionism (compared to the fit ideal) (McComb & Mills, 2022a), B/W multiracial women who endorse socially reinforced body ideals such as the curvy ideal may be at higher risk of developing maladaptive orthorexia symptoms. Additionally, women who report moderate to high levels of physical appearance perfectionism experience greater weight and appearance dissatisfaction and lower body satisfaction when socially comparing their appearance to the slim–thick ideal (compared to the thin ideal, fit ideal, or control conditions) (McComb & Mills, 2022b). This suggests that the curvy/slim–thick ideal may be particularly harmful to women’s embodiment experiences and may facilitate unhealthy body-altering eating practices to manage weight and shape concerns. Therefore, it is essential to explore the unique intersectional experiences of B/W multiracial individuals related to encounters of appearance-based racial identity invalidation and body ideal pressures, in addition to their association with problematic eating practices such as orthorexia. Doing so will also contribute to advancing sociocultural models of risk for maladaptive orthorexia attitudes and behaviors and add to the limited empirical literature examining orthorexia nervosa symptoms in racially diverse groups inclusive of multiracial individuals (see Arreguin et al., 2024).

### 1.3. The Present Study

Multiracial individuals are 30% more likely to have a mental health disorder than any other racial group (Miller et al., 2019). However, research focused on multiracial individuals’ experiences and their health is limited (Soltis & Barnes, 2025). Moreover, most research on disordered eating and body image centers on young adults (Costa et al., 2017; Samuels et al., 2019). Therefore, we examined the associations between racial identity invalidation, curvy ideal internalization, and maladaptive orthorexia symptoms and the indirect effects of racial identity invalidation on maladaptive orthorexia symptoms via curvy ideal internalization in a sample of B/W multiracial women between the ages of 18 and 61 living in the US. It is important to acknowledge that the current study only measured the curvy ideal internalization to focus on B/W multiracial women’s internalization of culturally specific appearance ideals rather than Western ideals focused on thinness and extend the literature on appearance ideals specific to their racialized identity. An exploratory aim was to evaluate whether a particular dimension of curvy ideal internalization would emerge as the strongest potential explanatory variable in the relationship between racial identity invalidation and maladaptive orthorexia symptoms in the sample. Additionally, the differential effects of racial identity invalidation frequency and racial identity invalidation distress in analyses is exploratory in nature.

**Hypothesis** **1.** *Racial identity invalidation will be positively correlated with maladaptive orthorexia symptoms and curvy ideal internalization.*

**Hypothesis** **2.** *Maladaptive orthorexia symptoms will be positively correlated with curvy ideal internalization.*

**Hypothesis** **3.** *Racial identity invalidation will demonstrate significant indirect effects on maladaptive orthorexia symptoms via curvy ideal internalization.*

## 2. Materials and Methods

### 2.1. Eligibility Criteria and Procedures

The current study utilized secondary data from a thesis project that examined the potential indirect effects of multiracial identity invalidation on mindfulness-based approaches to eating and exercise via positive embodiment experiences among B/W multiracial women (Pringle, 2025). Data from this parent thesis study were recently published (Pringle et al., 2026), and the only overlapping primary variables between that analysis and the present one include the racial identity invalidation measures. Methodology from the published parent thesis study is consistent with that of the current study. Before recruitment, the study received human-subjects approval from the UNC Charlotte IRb (#IRB–23-0747). Eligible participants identified as a B/W multiracial, cisgender female, over the age of 18, not currently pregnant, and English-speaking. Participants were provided with an online consent form as part of the initial Qualtrics survey and indicated consent before continuing to the measures. Three attention check items were dispersed randomly throughout the survey. The survey was administered through Prolific, a self-service questionnaire recruitment and administration platform. The survey took approximately 30 min to complete. Upon completion of the survey, participants were taken to a study debriefing page and provided with various mental health resources. Additionally, after completing the questionnaire and passing 2 out of 3 attention checks, participants received $6 as an incentive for taking part in the study based on a rate of $12/h, which exceeds the US federal minimum wage of $7.25/h. Data were collected between April and July 2024.

#### Participants

Participants included 290 (*M_Age_* = 34, *SD* = 9.7) B/W multiracial women between 18 and 61 who resided in the United States. More than half of participants reported that they do not strongly identify with being a Black biracial woman (56.3%). However, 30.8% described their racial identity as moderately important to them, and 60.3% racially identified as a mixed person. Based on their physical appearance, 70.5% of the sample considered themselves able to pass as multiple racial or ethnic groups. Most participants are sometimes perceived correctly as the race they are (65.8%), with 45.8% always being correctly perceived as a person of color. For individuals whose race is sometimes or hardly ever perceived correctly (not Black or African American, White or multiracial; 30.5%, respectively), most indicated that they are perceived to be of Hispanic/Latinx descent.

### 2.2. Measures

#### 2.2.1. Maladaptive Orthorexia Symptoms

The Teruel Orthorexia Scale (TOS; Barrada & Roncero, 2018) is a 17-item bidimensional measure assessing two related yet distinct constructs: healthy orthorexia and orthorexia nervosa (r = 0.43). Participants respond to each item on a 4-point Likert scale ranging from *completely disagree* (0) to *completely agree* (3). The healthy orthorexia construct (nine items, α = 0.80–0.85) aims to capture non-pathological interest in diet and healthy eating habits, whereas the orthorexia nervosa construct (eight items, α = 0.81–0.90) assesses pathological preoccupation with healthy eating through social and emotional impact. Sample items include “I mainly eat foods that I consider to be healthy,” and “Thoughts about healthy eating do not let me concentrate on other tasks.” Only the orthorexia nervosa construct scale was included in the current analysis. The orthorexia nervosa subscale score is calculated by summing the items, with higher scores indicating greater endorsement of maladaptive orthorexia symptoms. The internal consistency reliability of the current sample was α = 0.88.

#### 2.2.2. Racial Identity Invalidation

The Racial Identity Invalidation Scale (α = 0.95; Franco & O’Brien, 2018) is a 12-item measure that assesses multiracial individuals’ experiences of racial identity invalidation. This is understood through three forms: behavioral invalidation (invalidation based on nonstereotypical actions or behaviors), phenotype invalidation (invalidation based on appearance), and identity-incongruent discrimination (invalidation through misclassification into racial category one does not identify with). Sample items include “My physical features (e.g., skin color, hair texture, eye shape, eye color) lead people to assume that I am not the race(s) that I perceive myself,” and “Others think that my interests are different than those of a typical member of my racial group(s).” Participants responded on a 6-point Likert scale based on frequency ranging from *never* (1) to *almost always* (6), as well as how distressed they were by the experience ranging from *not at all distressed* (1) to *extremely distressed* (6). The full-scale score is calculated by averaging all items, with higher scores indicating greater experiences and distress related to racial invalidation. The internal consistency for the current sample was α = 0.87 (frequency scale) and α = 0.92 (distress scale).

#### 2.2.3. Curvy Ideals Internalization

The Curvy Ideals Internalization Scale (CIIS; Walker et al., 2022) is an 11-item measure containing three subscales: the internalization of generalized thick/curvy body ideal, as well as desire for a small/hourglass waist and large breasts as part of the ideal body composition (α = 0.80–0.90). Participants respond on a 5-point Likert agreement scale ranging from *strongly disagree* (1) to *strongly agree* (5). Sample items include “I prefer a fuller body shape,” “Having a small waist is important to me,” and “A desirable body has a large bust.” Notably this scale conceptualizes two separate curvy ideals: the thick ideal and slim–thick ideal. The full-scale and subscale scores are calculated by averaging all items, with higher scores indicating greater internalization of the curvy ideal. In the current sample, the internal consistency for the total scale was α = 0.86. The thick ideal subscale was α = 0.86, the slim–thick ideal subscale was α = 0.79, and the breast size subscale was α = 0.84.

### 2.3. Data Analysis Plan

Statistical analyses were conducted using the Statistical Package for the Social Sciences (SPSS), version 28 (IBM Corp, 2021). Descriptive statistics were performed with the demographic information and variables of interest on B/W multiracial women to assess whether the data fit a normal distribution to validate the use of parametric statistical tests. Statistical significance was determined as *p* < 0.05 for each analysis conducted. Percentages of missing data were calculated at the variable level. Little’s Test was used to evaluate patterns of missingness at the item level across measures to aid in informing the most appropriate approach to handling missing data (e.g., listwise deletion, all available data, multiple imputation, etc.) (Parent, 2013).

Pearson’s bivariate correlations were computed to investigate the first aim of the study, evaluating the basic linear relationships among racial identity invalidation, curvy ideal internalization, and maladaptive orthorexia symptoms. The effect size of the correlation coefficients were interpreted as small (*r* = 0.10), moderate (*r* = 0.30), or large (*r* = 0.50) based on Cohen’s criteria (Cohen, 1988).

For the second aim of the study, Hayes (2013) PROCESS macro model 4 for SPSS was used to detect the potential predicted indirect effects of racial identity invalidation (frequency and distress) on maladaptive orthorexia symptoms via curvy ideal internalization using multiple regression of the z-scores for each variable in four separate models (i.e., one with racial identity invalidation frequency on maladaptive orthorexia symptoms via curvy ideal internalization—global, and one with racial identity invalidation distress on maladaptive orthorexia symptoms via curvy ideal internalization—global, in addition to one model with racial identity invalidation frequency on maladaptive orthorexia symptoms via curvy ideal internalization—thin waist, thick/curvy, and breast size, and one with racial identity invalidation distress on maladaptive orthorexia symptoms via curvy ideal internalization—thin waist, thick/curvy, and breast size). Indirect effect analyses further computed contrasts between the magnitudes of pairs of mean indirect effects in multiple-mediator models of curvy ideal internalization—thin waist, thick/curvy, and breast size, and produced 95% CI’s surrounding these differences. The indirect effect models were not adjusted for covariates such as age due to the smaller concentration of participants that identified as middle adulthood and up. Additionally, perceived weight status and additional covariates were not included because this was not a priori, and post hoc analyses were not added to the current study as it was not powered to include testing several covariates. Z-scores were computed for all variables to facilitate the interpretation of the regression coefficients across measurement instruments. This mean-centering approach is used to rescale variables relative to their means and standard deviations to better understand how unusual or typical a particular measurement is compared with the overall distribution. A 10,000 non-parametric bootstrap resampling technique was used as a robust method for statistical inference to estimate the sampling distribution and confidence interval of the indirect effects. It yields an indirect effect beta coefficient estimate alongside a bias-corrected 95% confidence interval (CI). Beta coefficients are interpreted as the average change in standard deviations of maladaptive orthorexia symptoms associated with a one standard deviation change in curvy ideal internalization while holding other variables constant. Statistical significance is demonstrated when the 95% CI excludes zero (Hayes, 2013). These analyses were used to clarify whether experiences of racial identity invalidation indirectly affect maladaptive orthorexia symptoms via curvy ideal internalization in this age-diverse sample of B/W multiracial women.

## 3. Results

### 3.1. Data Screening

In the original study (Pringle, 2025), the survey received a total of 572 responses. Several responses were removed for not meeting inclusion criteria and/or not agreeing to participate in the survey, in addition to taking 80 min or more to complete the survey due to Prolific’s regulations (N = 234). Additional participants’ data were removed from the current analytic sample for failing to pass two out of three attention check items and progress to variable measure items (N = 14). This resulted in an analyzable sample of 324 participants.

Missing data were minimal across the primary study variables, ranging from 1.5% to 2.2%. Data were shown to be missing completely at random (Little’s chi-square test: X^2^ (1060) = 1132.3, *p* > 0.06). Therefore, a complete case analysis method was used for handling missing data (Parent, 2013). This resulted in a final analytic sample of 290. Table 1 contains select sociodemographic characteristics of the total sample.

### 3.2. Descriptive Statistics and Correlation Analysis

Table 2 presents the bivariate correlational findings and descriptive statistics for the primary study variables. When examining bivariate correlations, maladaptive orthorexia symptoms demonstrated a small yet significant positive correlation with racial identity invalidation frequency and racial identity invalidation distress. Additionally, small yet significant positive correlations were observed between maladaptive orthorexia symptoms and all curvy ideal internalization scales (i.e., global, thin waist, thick/curvy, and breast size subscales). Racial identity invalidation distress demonstrated small positive correlations with curvy ideal internalization—global, thick/curvy, and breast size subscales. Moreover, racial identity invalidation frequency was positively correlated with curvy ideal internalization—breast size, with observed estimates small in size. Notably, racial identity invalidation frequency demonstrated non-significant correlations with most curvy ideal internalization scales (i.e., global, thin waist, thick/curvy). Furthermore, racial identity invalidation distress and curvy ideal internalization—thin waist were not significant.

### 3.3. Indirect Effect Analysis

The Hayes (2013) PROCESS macro model 4 for SPSS was applied separately to each model. Models included racial identity invalidation frequency on maladaptive orthorexia symptoms via curvy ideal internalization—global; racial identity invalidation distress on maladaptive orthorexia symptoms via curvy ideal internalization—global; racial identity invalidation frequency on maladaptive orthorexia symptoms via curvy ideal internalization—thin waist, thick/curvy, and breast size; and racial identity invalidation distress on maladaptive orthorexia symptoms via curvy ideal internalization—thin waist, thick/curvy, and breast size. Visual inspection of the outputs confirmed consistency in the magnitude and directionality of estimates generated in both models.

**Figure 1:**
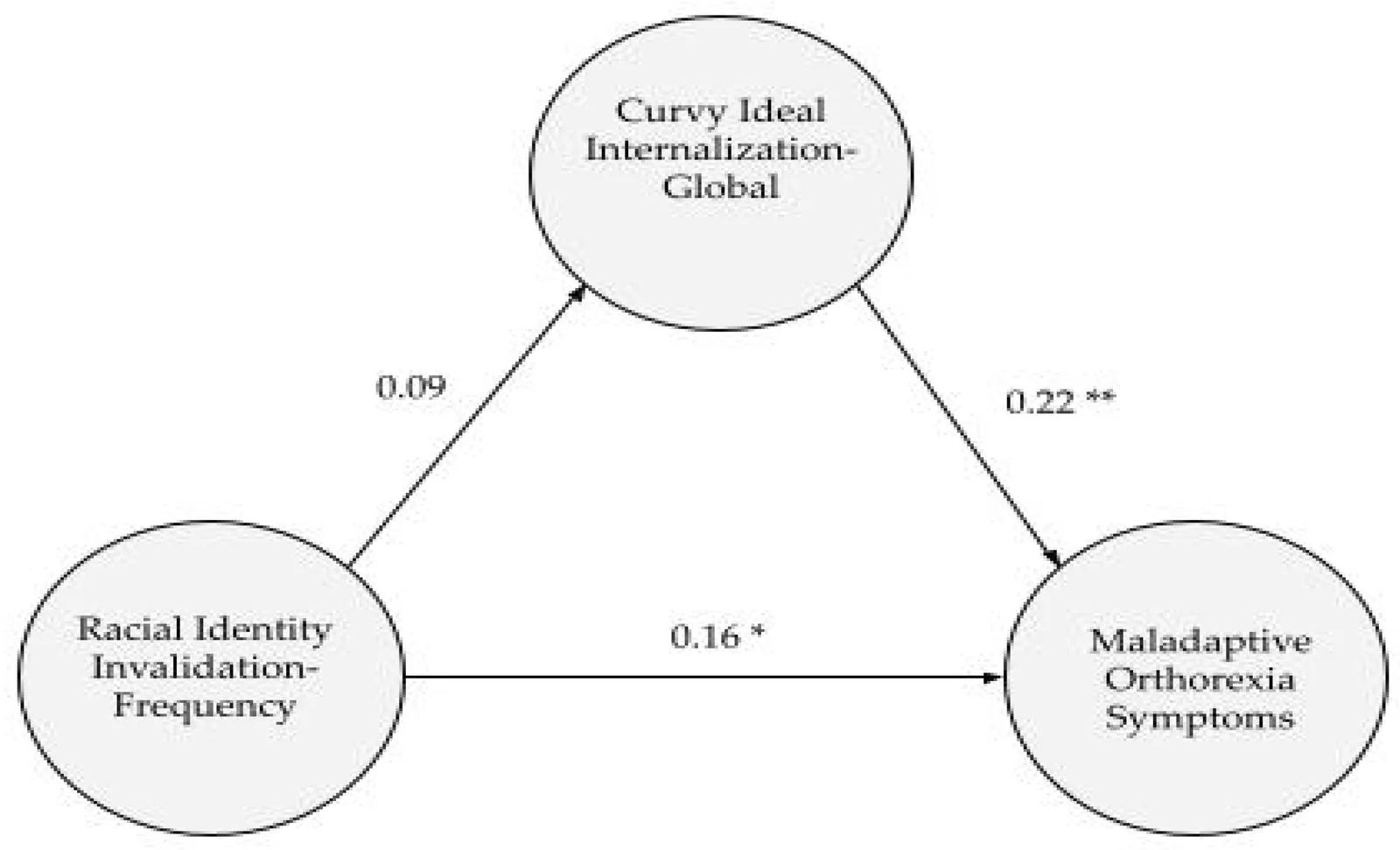
Indirect effect model of racial identity invalidation frequency on maladaptive orthorexia symptoms via curvy ideal internalization—global. *N* = 290. * *p* < 0.05; ** *p* < 0.01. All coefficients are standardized. Indirect effect of RII-F via CII-G: β = 0.02 (95% CI: −0.01, 0.05).

**Figure 2:**
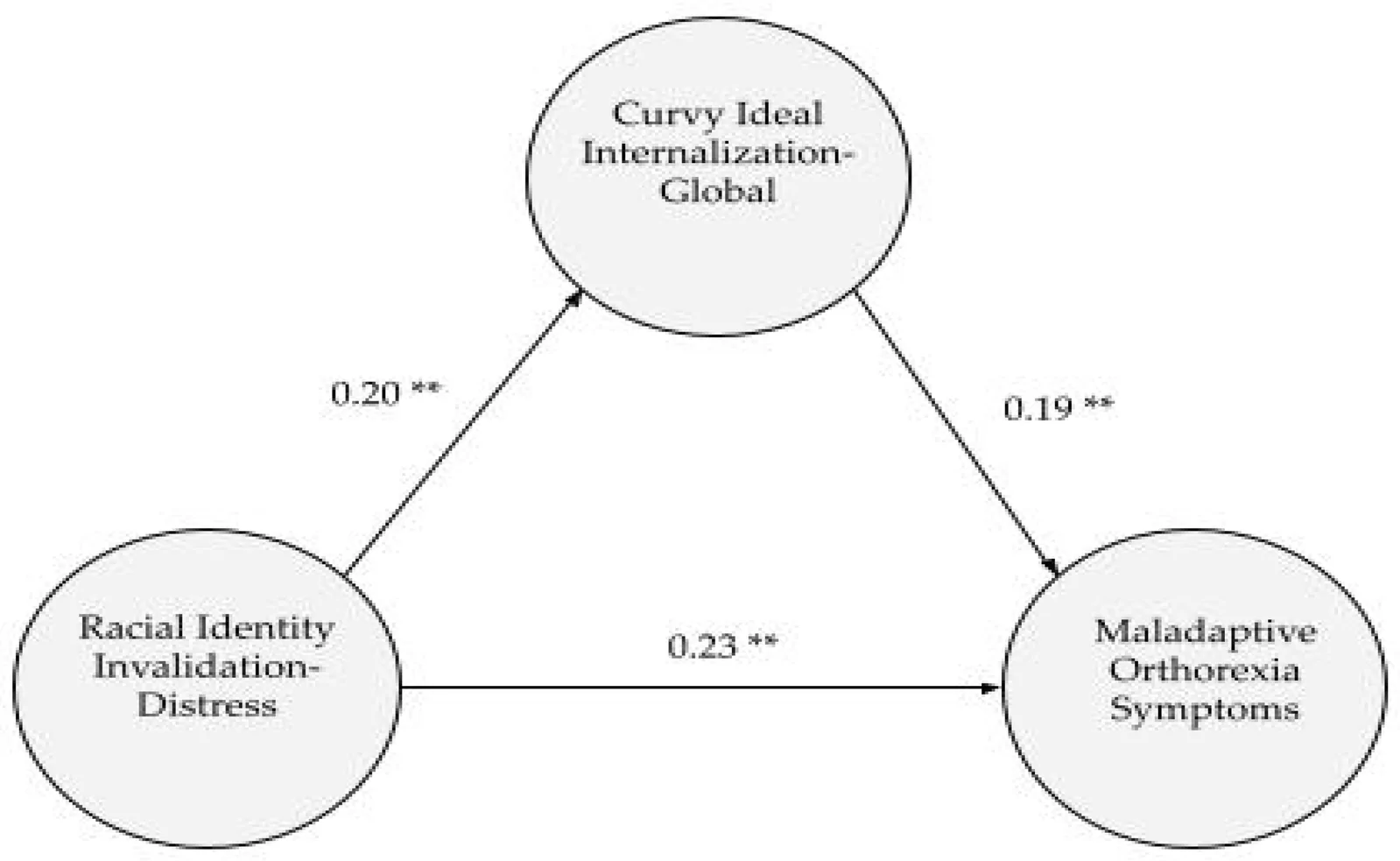
Indirect effect model of racial identity invalidation distress on maladaptive orthorexia symptoms via curvy ideal internalization—global. *N* = 290. ** *p* < 0.01. All coefficients are standardized. Indirect effect of RII-D via CII-G: β = 0.04 (95% CI: 0.01, 0.07).

**Figure 3:**
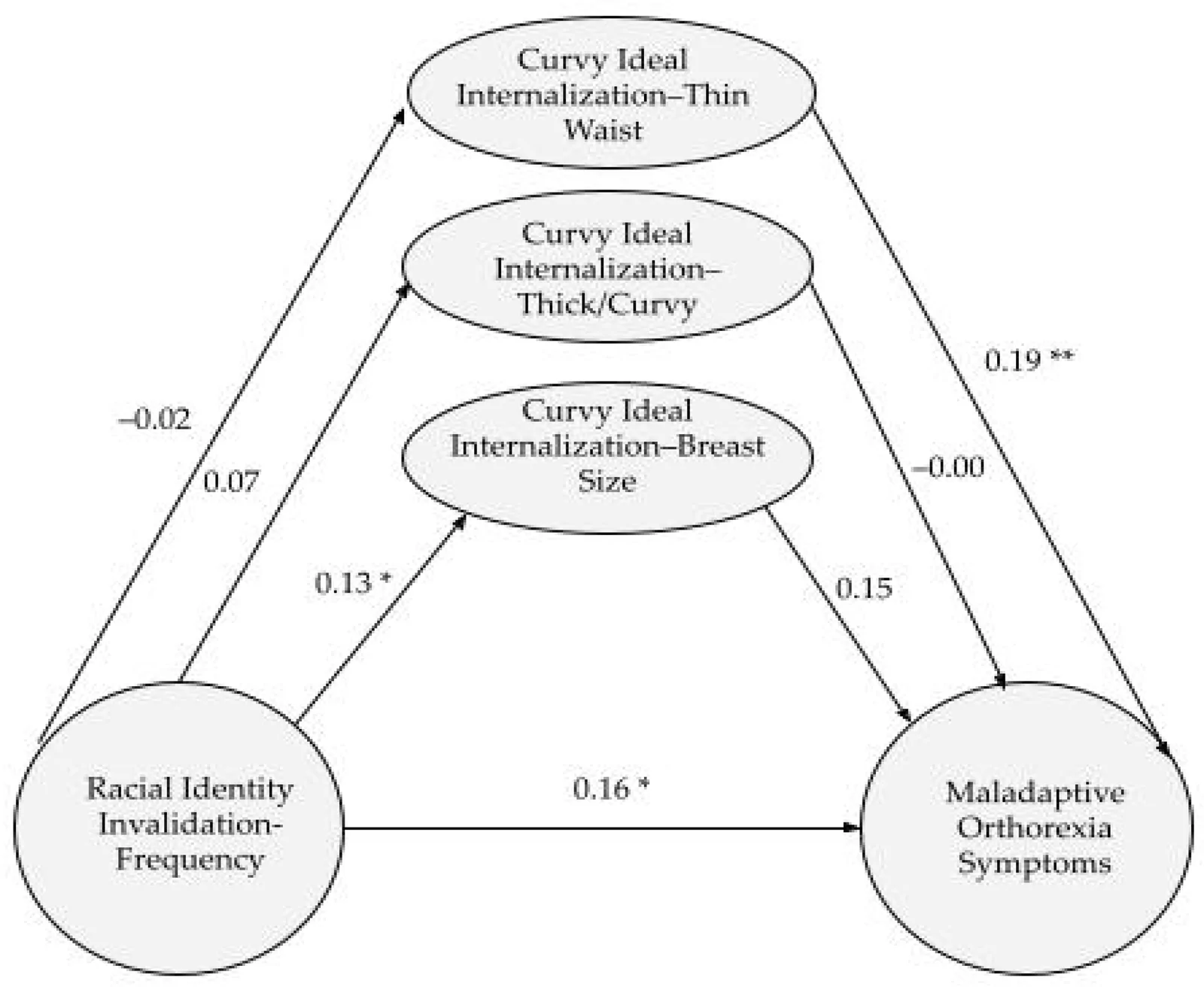
Indirect effect model of racial identity invalidation frequency on maladaptive orthorexia symptoms via curvy ideal internalization—thin waist, thick/curvy, and breast size. *N* = 290. * *p* < 0.05; ** *p* < 0.01. All coefficients are standardized. Indirect effect of RII-F via CII—thin waist: β = −0.00 (95% CI: −0.03, 0.03); CII—thick/curvy: β = 0.00 (95% CI: −0.02, 0.01); and CII—breast size: β = 0.02 (95% CI: −0.00, 0.05).

**Figure 4:**
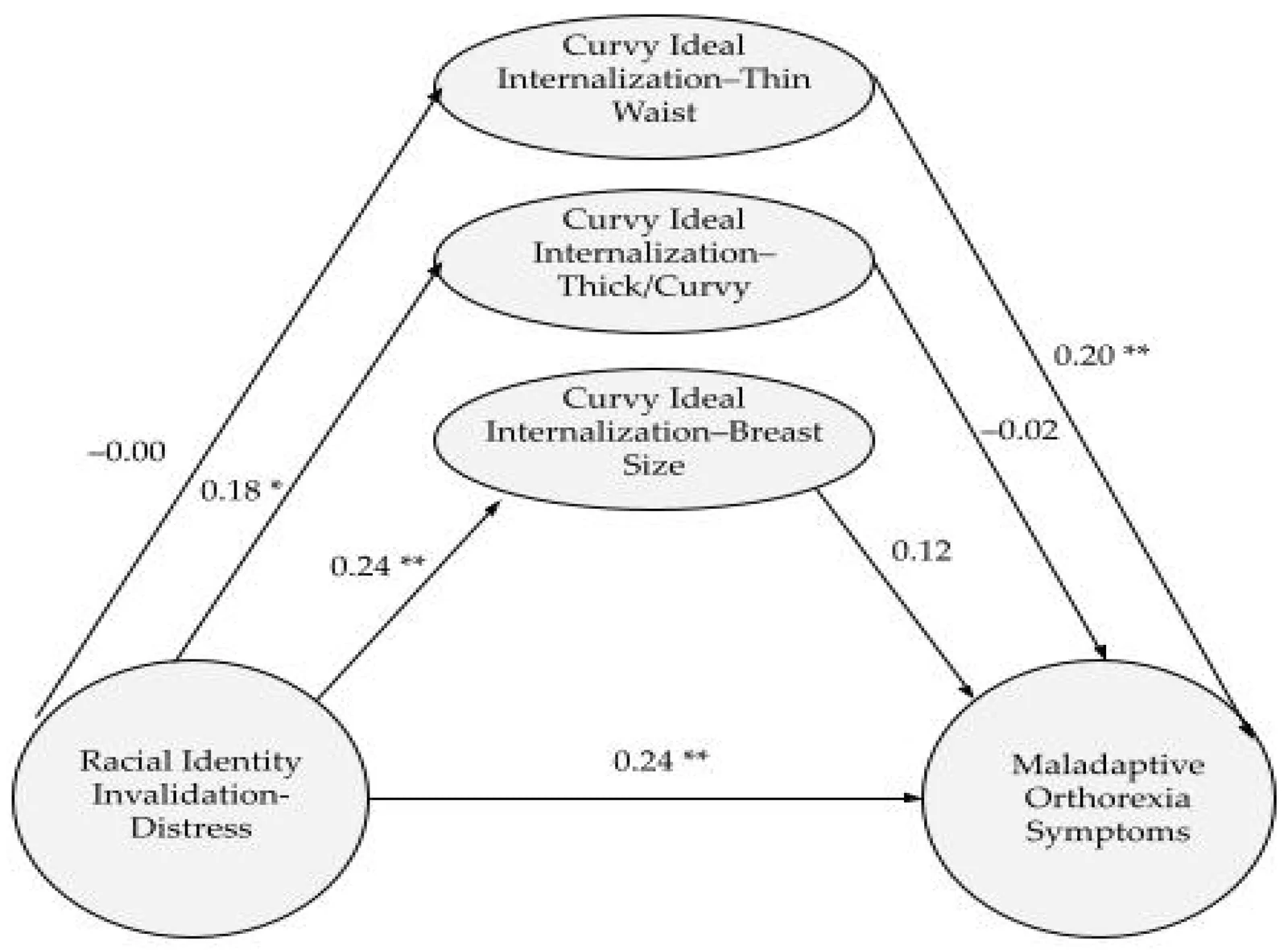
Indirect effect model of racial identity invalidation distress on maladaptive orthorexia symptoms via curvy ideal internalization—thin waist, thick/curvy, and breast size. N = 290. * *p* < 0.05; ** *p* < 0.01. All coefficients are standardized. Indirect effect of RII-D via CII—thin waist: β = 0.00 (95% CI: −0.03, 0.03); CII—thick/curvy: β = −0.00 (95% CI: −0.03, 0.02); and CII—breast size: β = 0.03 (95% CI: −0.00, 0.07).

In terms of variance accounted for, the racial identity invalidation frequency, curvy ideal internalization—global, and curvy ideal internalization—thin waist subscales explained 8% of the variance in maladaptive orthorexia symptoms scores (*p* < 0.001), whereas racial identity invalidation—distress accounted for 11% of the variance in maladaptive orthorexia symptoms scores (*p* < 0.001). The racial identity invalidation frequency and curvy ideal internalization—thick/curvy subscales explained 4% of the variance in maladaptive orthorexia symptoms scores (*p* < 0.001), while racial identity invalidation distress accounted for 8% (*p* < 0.001). The indirect effect of racial identity invalidation distress on maladaptive orthorexia symptoms via curvy ideal internalization—global was the only model that emerged as significant (β = 0.04, 95% CI: 0.01 to 0.07). Moreover, when compared in a combined, multiple-indirect-effect model, neither the curvy ideal internalization—thin waist, curvy ideal internalization—thick/curvy, nor the curvy ideal internalization—breast size subscales emerged as a stronger contributor in the indirect effect of racial identity invalidation on maladaptive orthorexia symptoms (please refer to Table 3 for complete regression information for indirect analyses). Indirect effect models for racial identity invalidation frequency and distress on maladaptive orthorexia symptoms via curvy ideal internalization—global are shown in Figure 1 and Figure 2, respectively. Additionally, indirect effect models for racial identity invalidation frequency and distress on maladaptive orthorexia symptoms via curvy ideal internalization—thin waist, thick/curvy, and breast size are shown in Figure 3 and Figure 4, respectively. Table 3 contains the complete regression analyses.

## 4. Discussion

Mobilized by recent expert calls to action (Landor et al., 2024; Lowy et al., 2021; Ramseyer Winter et al., 2026; Rodgers et al., 2025), the present study aimed to explore the relationships among experiences of racial identity invalidation, multiple facets of curvy ideal internalization, and endorsing maladaptive orthorexia attitudes and practices in an age-diverse community sample of B/W multiracial women. Addressing this aim was especially timely and important given the rapidly increasing representation of multiracial individuals in the US, evidence of this target population’s heightened vulnerability for negative embodiment (e.g., disordered eating behaviors) likely due to the intersectional appearance pressures inherent in gender identity socialization, and the scarce existing scholarship that has specifically explored the role of more unique, appearance-relevant racialized stressors faced by multiracial women in this context. Additionally, although the science supporting the adverse correlates and impacts of orthorexia nervosa is steadily accruing, there is a need to better situate exploring its potential sociocultural risk factors within broader bioecological frameworks (Landor et al., 2024; Meyer, 2003; Rodgers et al., 2025), which in turn hold promise for identifying multilevel targets for wellness promotion and harm reduction. Overall, the results were mixed in their consistency with our a priori predictions.

First, bivariate correlations revealed a pattern of findings mostly in accordance with our initial hypotheses. As anticipated, maladaptive orthorexia symptoms were positively associated with both dimensions of racial identity invalidation and all measures of curvy ideal internalization, although the effects were modest in magnitude. These results are consistent with previous research conducted with racially and ethnically diverse US (Hernández et al., 2021) and Canadian (McComb & Mills, 2022a) college women, indicating higher levels of disordered eating among participants endorsing greater aspirations to achieve some variant of the curvy ideal. The results also correspond with research highlighting racial discrimination as an influence on maladaptive eating behaviors among Black emerging adult women (Brown et al., 2022) and with recent quantitative reviews synthesizing the scholarship examining the links between discrimination and disordered eating in the broader literature (Mason et al., 2021; Rodrigues et al., 2022). At the same time, dimensions of racial identity invalidation were differentially related to components of curvy ideal internalization in the sample, with the distress measure demonstrating small, positive associations spanning most of the curvy ideal internalization variables. These findings suggest that distress appraisal may be more relevant than the mere frequency of exposure to these stressors in this context. For example, the frequency of invalidation may not necessarily denote adverse psychological and well-being outcomes; rather, distress related to invalidation may be more strongly associated with negative consequences (Franco & O’Brien, 2018). Moreover, factors such as racial identity salience/sense of racial identity and parental racial socialization may moderate the link between racial identity invalidation frequency and distress (Franco & Franco, 2016; Franco & O’Brien, 2018). The distress appraisal of invalidation, compared to frequency, may reflect a B/W multiracial woman’s feelings of lack of belonging to their racial identification (Franco & O’Brien, 2018) or harmful internalized interpretations attributed to the invalidation. However, it is important to note that the current analysis did not directly compare racial identity validation frequency and distress as a stronger predictor variable in analyses. Thus, future research would benefit from direct comparisons of racial identity validation frequency and distress on its association with racial identity strength, embodiment experiences, and health outcomes in B/W multiracial women. Future research should also explicitly explore the relationships between racial identity invalidation distress, lack of belonging based on one’s racial identity, internalized interpretations attributed to the invalidation, and embodiment experiences in Black multiracial women. Notably, other authors have also cited the positive associations between discrimination-related stress and body image in Latinas (Velez et al., 2015). Taken together, these initial-stage findings continue to support adopting a more critical and nuanced lens to challenge prevailing notions that substituting one unattainable idealized appearance standard (e.g., thinness) for a more culturally resonant one (e.g., curvaceousness) is not without associated health risks for women of color (Hughes, 2021; McElroy, 2025).

Second, building upon an examination of basic linear associations, the indirect effect model findings were partially consistent with our original predictions. The results indicated that a holistic assessment of curvy ideal internalization (rather than its fragmented components) may be a candidate psychological mechanism partially explaining how the distress engendered by experiences of racial identity invalidation exerts its influence on promoting preoccupation with and pursuit of orthorexia nervosa tendencies. This finding complements recently published evidence drawn from the same analytic sample which demonstrated a significant indirect effect of this racialized form of appearance-related oppression on mindful eating via positive embodiment (Pringle et al., 2026). However, it is important to note that temporal precedence was not tested in the current analysis, in which alternative directional models are plausible (e.g., individuals with greater maladaptive orthorexia symptoms may be more observant to culturally salient body ideals and in turn may experience appearance-based racial identity invalidation as more distressing). Additionally, given the high correlations among the curvy ideal internalization subscales, an alternative explanation for these findings may be that a holistic assessment of curvy ideal internalization may reflect greater reliability or variance captured by the composite (global) score rather than evidence that the global score is a more meaningful psychological mechanism. That is, the global construct’s conceptual and statistical overlap with the individual subscales makes it difficult to separate it as an independent construct in analyses. Thus, a holistic assessment may reflect the importance of recognizing the complexities and variance of experiences of curvy ideal internalization as an important mechanism in its association with racial identity invalidation distress and maladaptive orthorexia symptoms in B/W multiracial women. Collectively, this nascent body of scholarship suggests that this distinct form of appearance-based multiracial stress could be linked to engagement in both adaptive and maladaptive eating through body image- and embodiment-related variables. This interpretation a) aligns with existing minoritized stress (Meyer, 2003) and embodiment-centered (Landor et al., 2024; Piran & Teall, 2012; Rodgers et al., 2025) theoretical frameworks applied to a novel multiracial population and b) simultaneously expands traditional sociocultural models of disordered eating risk to consider the relevance of incorporating internalization measures reflecting greater diversity in appearance ideals (Alexander et al., 2024; Betz et al., 2019; Chilson et al., 2025; Hernández et al., 2021). This research further extends previous scholarship curating the psychosocial risk factors for orthorexia nervosa (McComb & Mills, 2019) and the limited research exploring orthorexia in minoritized groups inclusive of multiracial individuals (Arreguin et al., 2024; Bundros et al., 2016). Nevertheless, subsequent longitudinal or prospective analyses will be tasked with further clarifying or confirming these preliminary cross-sectional findings.

### Strengths, Limitations, and Future Research Directions

The present study adds to the scant literature exploring a minoritized stress/sociocultural model of maladaptive orthorexia attitudes and behaviors in a moderate-sized community sample of age-diverse B/W multiracial women. Preliminary findings may contribute to enhancing awareness among mental health clinicians, dietitians, and public health professionals of the importance of assessing the potential relevance of encounters with appearance-driven racial identity invalidation stress and the pursuit of the curvy ideal in the assessment of disordered eating practices such as orthorexia nervosa in this vulnerable population of multiracial women.

At the same time, there are important caveats to the current analysis that are offered as a springboard for future scholarship to address. Although these initial results are promising, the cross-sectional design precludes making conclusive interpretations about causality and the directionality of the relationships observed. Additionally, the measure of racial identity invalidation did not explicitly incorporate the intersectional experience of gender. Effect sizes were small and may be a function of the age range of the sample and variability in the salience or centrality of multiracial identity endorsed. Moreover, we exclusively investigated the potential effects of curvy ideal internalization in this context. Lastly, though justified, we centered the experiences of adult B/W multiracial women in this analysis.

In keeping with the above limitations, subsequent analyses are encouraged to implement prospective or longitudinal research designs to further examine the tenability and directionality of the associations among the variables featured in the models tested here. Additionally, future studies should consider placing greater attention to potential confounding variables given the possible influence of racial identity salience, age, etc., on appearance ideals, eating practices, and racial identity processes. Qualitative and mixed method approaches could additionally provide a deeper, multi-layered exploration of the lived experiences of B/W multiracial women navigating appearance-related stressors, pursuit of the curvy ideal, and orthorexic eating patterns. Incorporating a measure of gendered racial identity invalidation (e.g., gendered racial microaggressions: Volpe et al., 2024) and clarifying the perpetrators of racial identity invalidation (e.g., emotional/relational proximity) would advance our understanding of the embodiment-related impacts exacted by this unique form of intersectional appearance-based oppression that B/W multiracial women face. Meanwhile, experiences of colorism (Phoenix & Craddock, 2024) and internalized weight stigma (Ross et al., 2026) could be useful minoritized stressors to integrate into later-stage models. Testing for effect modifiers such as age or generation alongside the importance of multiracial identity could reveal relevant vulnerability or protective factors and is aligned with previous research demonstrating that the strength of racial–ethnic identity was associated with higher levels of curvy ideal internalization in Black and B/W biracial adult women (Keigan et al., 2024). Other potential individual difference factors future research may wish to explore in this context include adaptive self-regulatory processes like self-compassion (Volpe et al., 2024), multiracial identity pride (Christophe et al., 2021), and multiracial identity socialization experiences (Green et al., 2021). Comparing the effects of internalizing the curvaceous ideal with the internalization of other dominant appearance ideals (e.g., thin, athletic, muscular, etc.) would also be advantageous to pursue. Finally, future research warrants evaluating these and similar models across the wider spectrum of multiracial embodiment (e.g., adolescents, men, sexual and gender minorities, different combinations of multiracial identity, etc.).

## 5. Conclusions

In conclusion, the current analysis contributes to our understanding of B/W multiracial women’s sociocultural experiences and health, specifically related to their experiences of racial identity invalidation and its relationship with internalization of the curvy ideal and maladaptive orthorexia symptoms. Global curvy ideal internalization emerged as a candidate psychological mechanism in the relationship between racial identity invalidation distress and orthorexia nervosa tendencies. Future research is needed to further evaluate these associations using prospective or longitudinal research designs and including a more diverse multiracial sample (e.g., various racial combinations, gender identity, etc.) to better understand multiracial individuals’ discriminatory and embodiment experiences and their health. The results have implications for acknowledging the internalization of opposing cultural body ideals and experiences of distress related to discriminatory stressors as potential clinically relevant sociocultural factors associated with orthorexic eating patterns in B/W multiracial women.

## Figures and Tables

**Table 1:** Sociodemographic characteristics.

Sample Characteristic	Total (*n* = 295)
Age, years (mean and standard deviation)	34 (*SD* = 9.7)
**Perceived current weight**	
Underweight	7 (2.4%)
Somewhat underweight	16 (5.4%)
Just right	93 (31.5%)
Somewhat “overweight”	89 (30.2%)
“Overweight”	62 (21%)
Very “overweight”	28 (9.5%)
**Sexual orientation**	
Heterosexual	194 (66.2%)
Bisexual	45 (15.4%)
Lesbian	13 (4.4%)
Pansexual	16 (5.5%)
Queer	16 (5.5%)
Not listed	9 (3.1%)
**Currently living with a physical disability**	
Yes	32 (10.9%)
No	261 (89.1%)
**Highest level of education completed by mother**	
Never attended school or only attended kindergarten	1 (0.3%)
Grades 1 through 8 (elementary)	4 (1.4%)
Grades 9 through 11 (some high school)	12 (4.1%)
Grade 12 or GED (high school graduate)	84 (28.7%)
College 1 year to 3 years (some college or technical school)	85 (29%)
College 4 years (college graduate)	70 (23.9%)
Graduate school (advanced degree)	37 (12.6%)
**Current family household yearly income**	
Under $15,000	23 (7.8%)
$15,000–$25,000	28 (9.6%)
$25,000–$35,000	14 (4.8%)
$35,000–$50,000	52 (17.7%)
$50,000–$75,000	59 (20.1%)
$75,000–$100,000	45 (15.4%)
Above $100,000	72 (24.6%)
**Employment status**	
Employed full-time for wages	154 (52.2%)
Employed part-time for wages	63 (21.4%)
Not currently employed	76 (25.9%)
**Relationship status**	
Married	83 (28.1%)
Divorced	16 (5.4%)
Widowed	4 (1.4%)
Separated	6 (2%)
Single or never been married	130 (44.1%)
A member of an unmarried couple	56 (19%)

Note: N = 295. One to two missing data for some sociodemographic questions. Five participants were excluded for the final analyses due to missing data among study variables.

**Table 2:** Descriptive statistics and correlations among the primary study variables (*N* = 290).

	RII-F	RII-D	M-OS	CII-G	CII-TW	CII-T/C	CII-BS
*M*	3.1	2.7	13.8	3	3.9	3.8	2.3
*SD*	1	1.1	5.4	0.80	0.94	1.1	1.1
Range	1–6	1–6	8–30	1–5	1–5	1–5	1–5
RII-F		0.60 **	0.17 **	0.09	−0.01	0.08	0.13 *
RII-D			0.26 **	0.20 **	0.01	0.18 **	0.23 **
M-OS				0.23 **	0.22 **	0.13 *	0.22 **
CII-G					0.55 **	0.88 **	0.76 **
CII-TW						0.24 **	0.23 **
CII-BS							0.52 **

Note: * *p* < 0.05; ** *p* < 0.01. RII-F = racial identity invalidation frequency, RII-D = racial identity invalidation distress, M-OS = maladaptive orthorexia symptoms, CII-G = curvy ideal internalization—global, CII-TW = curvy ideal internalization—thin waist, CII-T/C = curvy ideal internalization—thick/curvy, CII-BS = curvy ideal internalization—breast size.

**Table 3:** Complete regression information for indirect analyses (N = 290).

95% Confidence Interval					
Effect	Estimate	SE	p	Lower	Upper
Path a					
RII-F → CII-G	0.0864	0.0589	0.1435	−0.0296	0.2024
RII-F → CII-TW	−0.0100	0.0588	0.8654	−0.1257	0.1057
RII-F → CII-T/C	0.0830	0.0584	0.1564	−0.0320	0.1980
RII-F → CII-BS	0.1320	0.0580	0.0237	0.0177	0.2462
RII-D → CII-G	0.1987	0.0581	0.0007	0.0843	0.3131
RII-D → CII-TW	0.0102	0.0586	0.8619	−0.1051	0.1255
RII-D → CII-T/C	0.1852	0.0579	0.0015	0.0713	0.2992
RII-D → CII-BS	0.2287	0.0570	0.0001	0.1165	0.3410
Path b					
(RII-F) CII-G → M-OS	0.2206	0.0571	0.0001	0.1082	0.3330
(RII-F) CII-TW → M-OS	0.2195	0.0564	0.0001	0.1085	0.3305
(RII-F) CII-T/C → M-OS	0.1171	0.0577	0.0431	0.0037	0.2306
(RII-F) CII-BS → M-OS	0.2003	0.0573	0.0005	0.0876	0.3130
(RII-D) CII-G → M-OS	0.1885	0.0572	0.0011	0.0759	0.3011
(RII-D) CII-TW → M-OS	0.2152	0.0553	0.0001	0.1064	0.3239
(RII-D) CII-T/C → M-OS	0.0850	0.0574	0.1400	−0.0281	0.1980
(RII-D) CII-BS → M-OS	0.1682	0.0575	0.0037	0.0551	0.2813
Total Effects—path c					
RII-F → M-OS (CII-G)	0.1756	0.0585	0.0029	0.0604	0.2907
RII-F → M-OS (CII-TW)	0.1715	0.0580	0.0034	0.0573	0.2856
RII-F → M-OS (CII-T/C)	0.1729	0.0578	0.0030	0.0591	0.2866
RII-F → M-OS (CII-BS)	0.1765	0.0578	0.0025	0.0628	0.2902
RII-D → M-OS (CII-G)	0.2694	0.0574	0.0000	0.1565	0.3823
RII-D → M-OS (CII-TW)	0.2596	0.0567	0.0000	0.1481	0.3711
RII-D → M-OS (CII-T/C)	0.2649	0.0569	0.0000	0.1530	0.3768
RII-D → M-OS (CII-BS)	0.2655	0.0566	0.0000	0.1540	0.3770
Direct Effects—path c’					
RII-F → M-OS (CII-G)	0.1565	0.0573	0.0067	0.0436	0.2693
RII-F → M-OS (CII-TW)	0.1737	0.0566	0.0024	0.0622	0.2851
RII-F → M-OS (CII-T/C)	0.1631	0.0577	0.0050	0.0496	0.2766
RII-F → M-OS (CII-BS)	0.1501	0.0572	0.0091	0.2627	0.1499
RII-D → M-OS (CII-G)	0.2320	0.0575	0.0001	0.1187	0.3452
RII-D → M-OS (CII-TW)	0.2574	0.0553	0.0000	0.1485	0.3663
RII-D → M-OS (CII-T/C)	0.2492	0.0577	0.0000	0.1355	0.3628
RII-D → M-OS (CII-BS)	0.2270	0.0574	0.001	0.1139	0.3401
Indirect Effects—path ab					
RII-F → M-OS via CII-G	0.0191	0.0149	-	−0.0083	0.0504
RII-F → M-OS via CII-TW	−0.0036	0.0145	-	−0.0331	0.0260
RII-F → M-OS via CII-T/C	0.0000	0.0067	-	−0.0157	0.0135
RII-F → M-OS via CII-BS	0.0204	0.0143	-	−0.0007	0.0543
RII-D → M-OS via CII-G	0.0374	0.0164	-	0.0105	0.0739
RII-D → M-OS via CII-TW	0.0000	0.0131	-	−0.0263	0.0277
RII-D → M-OS via CII-T/C	−0.0035	0.0137	-	−0.0333	0.0225
RII-D → M-OS via CII-BS	0.0296	0.0201	-	−0.0042	0.0746

Note: RII-F = racial identity invalidation frequency, RII-D = racial identity invalidation distress, M-OS = maladaptive orthorexia symptoms, CII-G = curvy ideal internalization—global, CII-TW = curvy ideal internalization—thin waist, CII-T/C = curvy ideal internalization—thick/curvy, CII-BS = curvy ideal internalization—breast size.

## Data Availability

De-identified data will be made available on request with appropriate ethics approval.
